# Supplementary material for: Identification of photoperiod-induced specific miRNAs in the adrenal glands of Sunite sheep (Ovis aries)
Source: Front Vet Sci. 2022 Jul 22;9:888207. doi: 10.3389/fvets.2022.888207 (PMC9354845; doi:10.3389/fvets.2022.888207)
Supplement: Supplementary file 1 [file Data_Sheet_1.ZIP › Supplementary file/miRDeep2 file.zip!/miRDeep2 file/Novel_Structure/LP42A1/Novel_515.pdf]

Provisional ID	:	NC_019483.2_34271
Score total	:	30.5
Score for star read(s)	:	3.9
Score for read counts	:	23.1
Score for mfe	:	1.9
Score for randfold	:	1.6
Score for cons. seed	:	
Total read count	:	57
Mature read count	:	56
Loop read count	:	0
Star read count	:	1

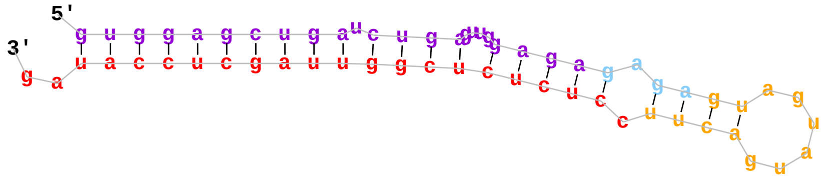

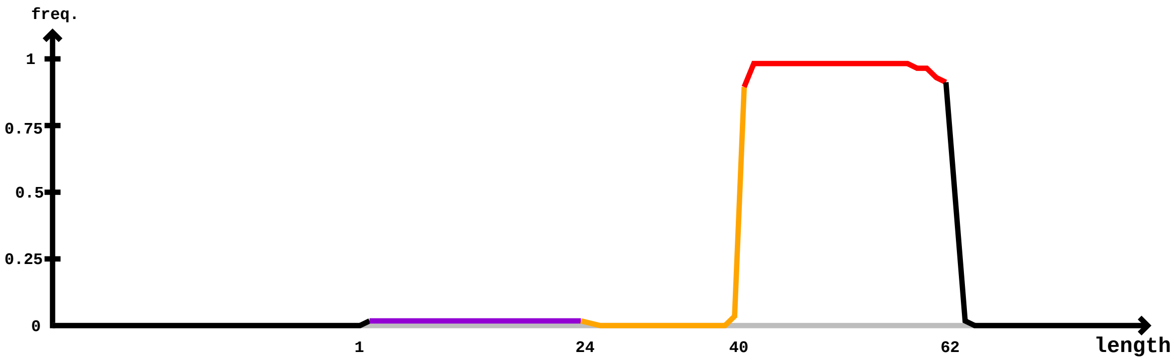

Star **Mature**

```
5'- uuuacucaguucacugaccgauugugccaguguggagcugaucugaguuggagagagaguaguaugacuuccucucucgguuagcuccauagauggcagaucagccuucucc   -3'    obs
    uuuacucaguucacugaccgauugugccaguguggagcugaucugaguuggagagagaguaguaugacuuccucucucgguuagcuccauagauggcagaucagccuucucc          exp
    .....((((....))))..((((.(((((.((((((((((.((((....(((((.((((......)))).)))))))))))))))))))..))))))))).........   reads     mm       sample
    ...............................guggagcugaucugaguuggaga..........................................................   1         0        seq
    .....................................................................uccucucucgguuagcuccauag....................   2         0        seq
    ......................................................................ccucucucgguuagcucc........................   1         0        seq
    ......................................................................ccucucucgguuagcuccau......................   2         0        seq
    ......................................................................ccucucucgguuagcuccaua.....................   1         0        seq
    ......................................................................ccucucucgguuagcuccauag....................   32        0        seq
    ......................................................................ccucucucgguuagcuccauaga...................   12        0        seq
    ......................................................................ccucucucgguuagcuccauagau..................   1         0        seq
    .......................................................................cucucucgguuagcuccauag....................   3         0        seq
    .......................................................................cucucucgguuagcuccauaga...................   2         0        seq
```
